# *Tridax procumbens*: Applicable Weed in Phytoremediation and Bioindication of Soil Contamination by Mercury

**DOI:** 10.1021/acsomega.5c03362

**Published:** 2025-06-17

**Authors:** Evandro Alves de Oliveira, Larissa Cavalheiro da Silva, Leandro Dênis Battirola, Ricardo Lopes Tortorela de Andrade

**Affiliations:** † 583431 Federal University of Mato Grosso, Sinop Campus; Institute of Natural, Human and Social Sciences Graduate Program in Environmental Sciences. 1200 Alexandre Ferronato Avenue, Industrial Sector, Sinop 78557-267, Mato Grosso, Brazil

## Abstract

Spontaneous plants have significant potential for phytoremediation of metal-contaminated soils. This study evaluated *Tridax procumbens L*. for mercury (Hg) decontamination. The plant was cultivated in a sand and vermiculite (1:1) substrate with Hg concentrations of 0, 1, 3, 5, and 7 mg kg^–1^. After 90 days, biometric growth and total Hg uptake by the aerial and root tissues were analyzed. Bioconcentration (BCF), translocation (TF), and bioaccumulation (BAF) factors, along with the elongation ratio (RER), tolerance (TI), and absorption (AI) indices, were used to assess the phytoremediation potential. BAF ranged from 1.46 to 11.04, BCF ranged from 6.07 to 30.31, and TF averaged 0.72, showing high Hg accumulation in roots and shoots. Vegetative development was affected by the concentration of Hg in the substrate (Hg_s_), with TI varying from 97 to 6.62% at Hg_s_ of 1 and 7 mg kg^–1^, respectively. AI increased proportionally to Hg_s_, ranging from 8.12 to 17.81 μg, indicating that the mass of Hg absorbed by the plant increased even with a reduction in biomass production. *T. procumbens*, despite the reduction in its biomass, did not show any visual signs of chlorosis or necrosis, demonstrating resistance to Hg toxicity. These findings suggest that *T. procumbens* is a promising phytoremediator for soil decontamination and bioindication of Hg contamination, contributing to global efforts to protect, restore, and promote the sustainable use of terrestrial ecosystems and to halt and reverse land degradation and biodiversity loss.

## Graphical abstract

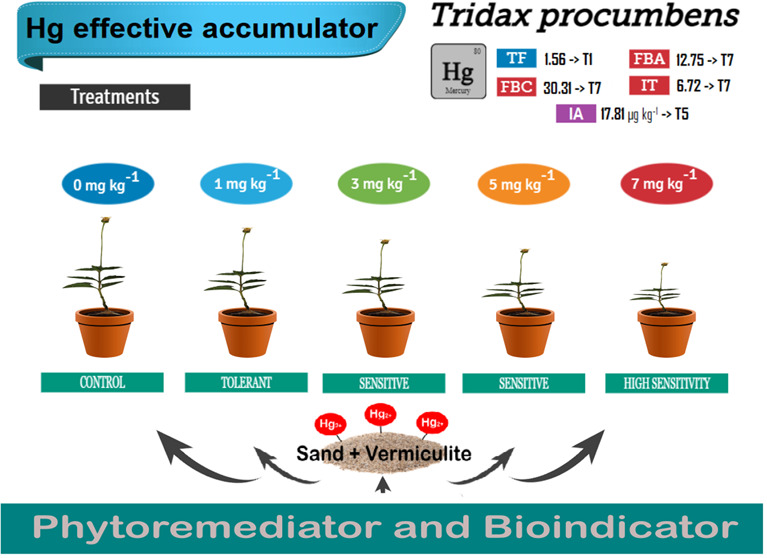

## 1 Introduction

Mercury (Hg) is a potentially dangerous contaminant due to its high toxicity, mobility, and biomagnification capacity, posing a significant risk to human health and the environment.^1–3^ The increase in the deposition of Hg in the soil, due to anthropogenic activities, has aroused growing global concern.^4^ High concentrations of mercury in the soil can inhibit crop growth or cause plant death, as well as directly harm human health due to the bioaccumulation process.^5^

In uncontaminated soils, background mercury concentrations typically range from 0.01 to 0.1 mg kg^–1^, while urban soils often exhibit higher levels due to anthropogenic inputs, reaching up to 1.5 mg kg^–1^ depending on industrial activity and atmospheric deposition.^6,7^ Although mercury is not essential for biological systems, it is highly toxic and persistent. Chronic exposure to elevated Hg levels can impair plant growth, disrupt microbial communities, and pose serious risks to human health through bioaccumulation and trophic transfer. Global anthropogenic mercury emissions are estimated at approximately 2220 t per year, contributing to widespread environmental contamination.^8,9^ Artisanal and small-scale gold mining is one of the main activities responsible for the release of Hg into the environment, and this practice is very common in South America, Africa, and Asia.^10,11^

Soil preservation and monitoring are crucial factors in maintaining environmental balance and guaranteeing the sustenance of life, as well as the development of economic, industrial, and agricultural activities.^12^ In addition to supporting productive functions, soil conservation plays a vital role in restoring degraded areas, preventing habitat loss, and safeguarding biodiversity, reinforcing its centrality in sustainable land use strategies.^13^

In this scenario, phytoremediation has emerged as a promising and effective approach to mitigating contamination of this habitat.^9^ This technique involves the use of plants capable of tolerating, accumulating, removing, or immobilizing contaminants present in the soil,^14^ different physiological processes of a given species can be used in the remediation process, in which phytoextraction and phytostabilization are the most commonly used.^15^

In this sense, spontaneous or pioneer plants are important for phytoremediation studies due to their favorable characteristics for this technique, such as high biomass production and the ability to develop under adverse conditions.^16,17^ In addition to remediation, these plants can also act as bioindicators of soil contamination, helping to assess the quality and levels of pollutants present.^18^ Spontaneous plants such as *Brassica juncea* (L.) Czern. (Brassicaceae),^19^ *Coincya monensis* (L.) (Brassicaceae),^20^ *Emilia fosbergii* Nicolson (Asteraceae)^21^ and *Paspalum fasciculatum* Willd (Poaceae)^22^ have been studied in Hg-contaminated environments, where they play crucial roles in phytoremediation by accumulating the metal (found therein)­and promoting the recovery of polluted soils.

An example of a very common pioneer species is *Tridax procumbens* L. (Asteraceae), originally from Central America and now widely distributed geographically. It is an annual herbaceous plant that forms dense clumps. The species exhibits a capitulum-type inflorescence typical of the Asteraceae family, consisting of central tubular florets surrounded by peripheral ligulate florets. Its fruits are cypselae, usually equipped with a pappus that facilitates wind dispersal, and contain lightweight seeds adapted to anemochory.^23,24^ In Brazil it is commonly found in the Midwest and Southeast regions, where it has intense and continuous blooms, high seed production and efficient dispersal, easy adaptation, and rapid growth. These characteristics of *T. procumbens* allow for it to be characterized as a ruderal plant, occurring on prairies, lawns, roadsides, and between rocks. In certain localities, it is used in traditional medicine.^25^

Previous findings have reported the occurrence of T. procumbens in Hg-contaminated urban soils, with concentrations of 51.5 μg kg^–1^ in roots and 27.4 μg kg^–1^ in shoots, and corresponding BCF and BAF values of 0.47 and 0.25, respectively, in soils averaging 108.8 μg kg^–1^ of Hg, which supports its potential for phytoremediation under controlled conditions.^26^ Considering the hardiness and wide occurrence of *T. procumbens*, this study evaluated its potential for phytoremediation of mercury-contaminated soils. To this end, the aim was to analyze the development, biomass production, extraction, and accumulation of mercury in its biomass at different levels of contamination under experimental conditions, contributing to the advancement of knowledge on the application of spontaneous plants in the phytoremediation and bioindication of Hg-contaminated soils, generating relevant information for the development of more efficient and sustainable environmental remediation strategies.

## 2 Material and Methods

### 2.1 Species Selection and Seed Collection

*Tridax procumbens* was selected for this study due to its wide occurrence and geographical distribution, showing good adaptability to varied habitat conditions, meeting criteria common to species suitable for phytoremediation processes, such as reproduction through seeds, high biomass production, rapid growth, easy adaptation to local soils and low management requirements.^16^

Seeds of *T. procumbens* were obtained from healthy adult plants found in the urban perimeter of Sinop-MT, in the south of the Brazilian Amazon. The region has an altitude of 380 m, with a hot and humid tropical climate (AW) according to the Köppen classification. The average annual temperature is 24.2 °C, with lows of 20 °C and highs of 33 °C.^27,28^ The *T. procumbens* seeds were selected so that they had uniform morphological characteristics and no traces of structural damage. In the laboratory, the seeds were washed and disinfected with 0.25% (w/v) sodium hypochlorite for 15 min, washed again in distilled water, separated, dried, and stored in sealed plastic containers until the moment of cultivation.^19^

#### 2.1.2 Substrate and Cultivation

The plants were grown in a greenhouse under natural environmental conditions. The substrate used consisted of 1 kg of a mixture of washed sand and vermiculite in a ratio of 1:1 (v/v).^29^ Sowing took place in identical individual pots filled with the substrate, with ten seeds distributed in each pot. The washed sand was sieved and sterilized in 0.25% (w/v) sodium hypochlorite.^30^ The growing period after germination was 90 days. Throughout cultivation, the plants were irrigated an average of four times a week, up to three-quarters of their field capacity, in order to avoid leaching of the contaminant.^31^ Clark’s nutrient solution was used to nourish the seedlings,^32^ with an ionic concentration of 70%, with applications on average every 15 days.

After germination, one healthy seedling was maintained per pot to ensure uniformity among replicates. Three independent pots per treatment were used, with each considered one experimental unit. The experimental design was entirely randomized and consisted of five different Hgs: 0, 1, 3, 5, and 7 mg kg^–1^, using mercury­(II) chloride as the source of Hg^2+^.

#### 2.1.3 Chemical Analysis

At the end of the growing period, samples of plant tissue were collected and taken to the laboratory for chemical analysis. Each sample unit was divided into aerial and root parts and then weighed (wet weight). After the initial weighing, the samples were transferred to an oven with forced air circulation and dried at 50 °C until a constant weight.

After drying, they were crushed, about 0.3 g (dry weight) was transferred to a digestion tube, and 1 mL of distilled water, 2 mL of a 1:1 ratio mixture of 65% nitric acid (HNO_3_), and 70% perchloric acid (HClO_4_) and 5 mL of 98% sulfuric acid (H_2_SO_4_) were added. The tubes remained in the digestion block at 230 °C for 30 min, then cooled to room temperature, transferred to 25 mL volumetric flasks, and diluted in distilled water.^33^

The concentration of Hg in the samples was assessed by cold vapor atomization atomic absorption spectroscopy (CV-AAS) on a Varian AA140 spectrometer coupled to a VGA 77 vapor generator accessory. The Hg standard solution used for calibration was traceable to NIST (National Institute of Standards and Technology), brand Specsol, with a concentration of 1000 mg L^–1^.

The method was validated according to Neto et al.^34^ The relative precision of ± 5.8% of the analytical method was determined for total mercury in leaves with samples fortified with three different concentrations and seven repetitions. The accuracy, determined by the recovery of Hg in the fortified samples, was between 95 and 110%. The detection limit, defined as the average of ten blanks plus three times the standard deviation, was 0.08 μg L^–1^ in the sample solution, or 6.7 μg kg^–1^ in dry biomass, and the quantification limit, defined as the average of ten blanks plus ten times the standard deviation, was 0.16 μg L^–1^ in the sample solution, or 13.3 μg kg^–1^ in dry biomass.

### 2.2 Data Analysis

#### 2.2.1 Plant Development and Tolerance

Plant growth was assessed using measurements of biomass produced, in grams of dry material, and root and aerial relative elongation ratios (RER), to estimate root growth and above-ground biomass growth, respectively.^35^ The plants’ root RER values were defined as the ratio between the average root length of the tested plant and the average root length of the control plant, and the aerial RER was defined as the ratio between the average length of the tested plant and the average length of the control plant.^36^

The tolerance index (TI) was used to measure plant biomass growth and tolerance to Hg and was determined by the ratio between the dry biomass of plants treated with Hg and the biomass of the control.^37^

#### 2.2.2 Phytoremediation Indices

Hg concentration and accumulation trends were estimated using the accumulation (BAF), translocation (TF), and bioconcentration (BCF) factors, calculated from the mercury concentrations determined from chemical analyses of the samples. The TF was calculated from the ratio between the Hg concentration values in the aerial parts of the plants and in the root part.^38^

BCF was determined from the value expressed as the ratio between the metal concentration in the root and the concentration in the substrate.^39^ The bioaccumulation factor (BAF) was determined by calculating the ratio between the concentration of the metal in the aerial part and the concentration of the metal in the substrate.^40^

The absorption index (AI) represents the mass of Hg accumulated by the plant tissues and was obtained from the product between the concentration of Hg in the tissue and the dry plant biomass produced, in kg.^41^

#### 2.2.3 Statistical Analysis

The analyses were carried out using the statistical language “R” (version 4.4.0, R Foundation for Statistical Computing) and RStudio (version 2024.4.2.764). We used the packages dplyr (1.1.4), tidyr (1.3.1), ggplot2 (3.5.1), gridExtra (2.3), multcompView (0.1–10), agricolae (1. 3–7), writexl (1.5.0), gt (0.10.1), tidyverse (2.0.0), officer (0.6.6), gapminder (1.0.0), flextable (0.9.6) and VennDiagram (1.7.3). A *p*-value <0.05 was considered statistically significant. Results are presented as mean ± standard deviation. The graphical presentation was carried out using OriginPro 2018b software. Where necessary, the data were subjected to analysis of variance (ANOVA) to check for differences between means, followed by the Scott-Knott test to compare means. The Shapiro-Wilk test was used to check for normality and, where necessary, the data were transformed to obtain a normal distribution. Pearson’s correlation was used to determine the relationship between the variables and the contamination applied.

## 3 Results

### 3.1 Plant Development and Tolerance

The effects of Hg contamination on the development of T. procumbens were assessed, with germination occurring first in the units with the lowest concentration of the contaminant. The germinated seedlings were not deformed, and there were no signs of chlorosis or necrosis in any of the sample units. There was a decrease in overall plant growth as a result of the increase in (ANOVA, F = 78.59 and *p* < 0.05), with a correlation coefficient of −0.96353, starting at 178.33 mm in the absence of the metal, and reaching 59 mm at Hgs of 7 mg kg^–1^ (Table 1). There was a strong negative correlation (*p* < 0.05) with the increase in Hg_s_, both for the aerial RER (r = −0.96) and the root RER (r = −0.87).

**Table 1:** Relative Elongation Ratio of Roots, Aerial Part, and Whole Plant, Showing Variation in Plant Elongation as a Function of Hg_s_ ^a^

	whole plant		aerial part			root part		
Hg_s_ (mg kg^–1^)	average length [mm]		average length [mm]		aerial RER [%]	average length [mm]		root RER [%]
0	178	±12 ^a^	81	±9 ^a^		96	±6 ^a^	
1	158	±6 ^b^	69	±3 ^b^	84.49	89	±3 ^a^	92.07
3	116	±12 ^c^	62	±6 ^b^	76.73	54	±6 ^b^	55.86
5	65	±10 ^d^	31	±4 ^c^	37.96	34	±12 ^c^	35.17
7	59	±10 ^d^	18	±1 ^d^	22.45	40	±8 ^c^	42.07

a Different lowercase letters in the columns indicate statistical differences using the Scott-Knott test (*p* < 0.05); equal letters do not differ significantly.

The Scott-Knott test showed that, for the whole plant, there were differences between all the treatments, except between the Hg_s_ values of 5 and 7 mg kg^–1^, which did not differ from each other. In the aerial part, the Hg_s_ of 1 and 3 mg kg^–1^ showed no significant difference, but differed from the other treatments, with the highest values observed in the control treatment (0 mg kg^–1^) and the lowest in the Hg_s_ of 7 mg kg^–1^. In the root part, the Hg_s_ of 0 and 1 mg kg^–1^ also did not differ from each other, but they did differ from the Hg_s_ of 3 mg kg^–1^, as well as from the Hg_s_ of 5 and 7 mg kg^–1^, which were the lowest values observed and did not differ from each other.

Biomass production was significantly affected by the increase in Hg concentration, reducing from 2.15 g of dry biomass in the absence of the metal to 0.14 g at an Hg_s_ of 7 mg kg^–1^ (Table 2). There were significant differences in total dry mass between the different Hgs (ANOVA, F = 39.7, *p* < 0.05), with a strongly negative correlation with the increase in Hg_s_, both in the aerial part (r = −0.95) and in the root (r = −0.86).

**Table 2:** Dry Mass, Expressed in Grams, Per Sample Unit of *T. procumbens* Collected after Cultivation in a Substrate Contaminated with Different Hg_s_ ^a^

Hg_s_ (mg kg^‑1^)	whole plant	aerial part	root part
0	2.15 ± 0.34 ^a^	1.59 ± 0.25 ^a^	0.56 ± 0.14 ^a^
1	2.09 ± 0.29 ^a^	1.40 ± 0.16 ^a^	0.69 ± 0.13 ^a^
3	1.02 ± 0.30 ^b^	0.71 ± 0.23 ^b^	0.31 ± 0.08 ^b^
5	0.60 ± 0.10 ^b^	0.28 ± 0.07 ^c^	0.33 ± 0.03 ^b^
7	0.14 ± 0.03 ^c^	0.10 ± 0.02 ^c^	0.05 ± 0.01 ^c^

a Different lowercase letters in the columns indicate statistical differences using the Scott-Knott test; equal letters do not differ significantly.

The Scott-Knott test showed that the means of the Hg_s_ of 0 and 1 mg kg^–1^ (2.15 and 2.09 g) are statistically equal to each other and higher than the means of the 3 and 5 mg kg^–1^ (1.02 and 0.60 g). The masses obtained at an Hg_s_ of 7 mg kg^–1^ (0.14 g) were significantly lower than all the others. For aerial dry mass, the averages of Hg_s_ 0 and 1 mg kg^–1^ (1.59 and 1.40) are equal to each other and higher than the average of Hg_s_ 3 mg kg^–1^ (0.71 g). The averages for Hg_s_ 5 and 7 mg kg^–1^ (0.28 and 0.10 g) are the lowest. In terms of root dry mass, the averages for Hg_s_ 1 and 0 mg kg^–1^ (0.69 and 0.56 g) are equal to each other and higher than the averages for Hg_s_ 5 and 3 mg kg^–1^ (0.33 and 0.31 g). The average Hg_s_ of 7 mg kg^–1^ (0.05 g) is the lowest.

There was an exponential decrease in TI as a function of the increase in Hgs, with a correlation coefficient of −0.93. The specimens grown with Hgs of 1 mg kg^–1^ had a tolerance index of 97% (Table 3), classifying the plant as tolerant to this concentration of metal.^37^ However, with the increase in Hg_s_, there was a significant reduction in the tolerance index, dropping to 47.49% at Hg_s_ of 3 mg kg^–1^, classifying it as sensitive, and reaching 6.72% for Hg_s_, of 7 mg kg^–1^, classifying it as “highly sensitive”. On average, the TI was 44.82%, classifying *T. procumbens* as a Hg-sensitive plant.

**Table 3:** Tolerance Index, Expressed in (%) Based on the Ratio between the Biomass of *T. procumbis* Grown in a Contaminated Substrate under Different Concentrations of Hgs and the Biomass of the Control^a^

	tolerance index (%)	
Hg_s_ (mg kg^–1^)	TI (%)	tolerance classification
1	97.00 ^a^	tolerant
3	47.49 ^b^	sensitive
5	28.08 ^c^	sensitive
7	6.72 ^d^	highly sensitive

a The tolerance index values classify the plants as highly sensitive (TI ≤ 25%), sensitive (TI between 25 and 50%), moderately tolerant (TI between 50 and 75%), tolerant (TI between 75 and 100%), and highly tolerant (TI > 100%). Different lowercase letters in the columns indicate statistical differences by the Scott-Knott test (*p* < 0.05); equal letters do not differ significantly.

### 3.2 Accumulation of Hg in Plant Tissues

There were significant differences in the concentration of accumulated Hg between the different Hg_s_ for all the parts analyzed (whole plant, ANOVA, F = 46.16, *p* < 0.05, root part, ANOVA, F = 14.16, *p* < 0.05 and aerial part, ANOVA, F = 30.64, *p* < 0.05). These results show that the treatments significantly influence the concentration of Hg in the different parts of the plant.

Figure 1 shows that the accumulation of Hg in *T. procumbens* increased exponentially with the increase in Hg_s_, with a correlation coefficient of 0.9817 for the whole plant, 0.9722 for the roots, and 0.8529 for the aerial tissues. The results indicate that the plant extracted and accumulated a large amount of the contaminant from the substrate, with concentrations reaching 49.46 mg kg^–1^ in the aerial part (Figure 1a) and 117.63 mg kg^–1^ in the root part (Figure 1b), both for the treatment with Hgs of 7 kg^–1^.

**Figure 1:**
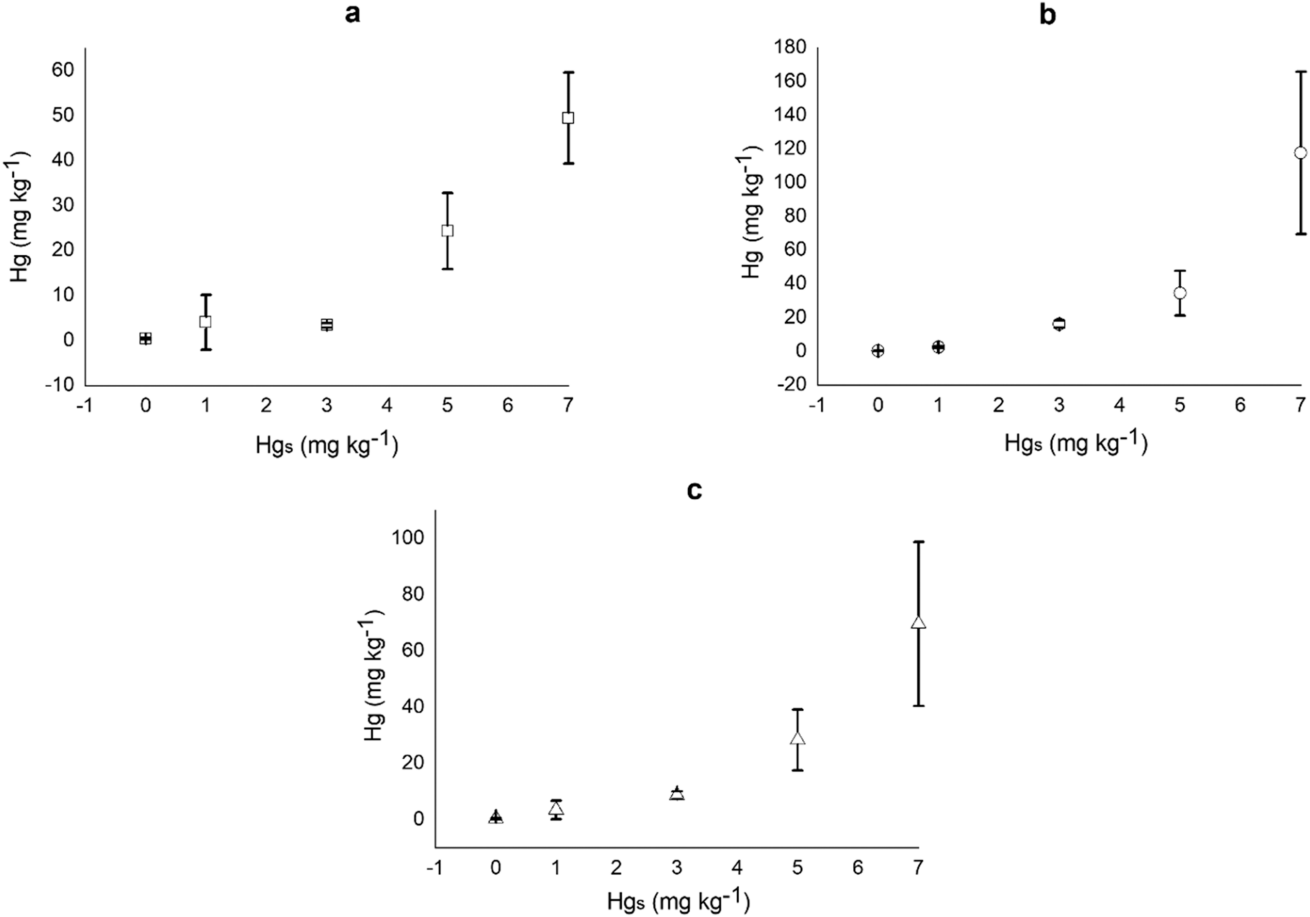
Hg concentration in different parts of the *Tridax procumbens* plant (aerial part (a) □, roots (b) △, and whole plant (c) ▲) in response to Hg concentrations applied to the substrate (0, 1, 3, 5, and 7 mg kg^–1^). Values are presented in milligrams kg^–1^ ± standard deviation.

The application of the Scott-Knott test for the concentration of Hg in the whole plant showed that the highest concentration was recorded in the treatment with 7 mg kg^–1^ (72.73 mg kg^–1^), followed by 5 mg kg^–1^ (29.49 mg kg^–1^), while the concentrations of 0, 1, and 3 mg kg^–1^ (0.37, 3.89, and 7.32 mg kg^–1^, respectively) were the lowest and did not differ significantly from each other. For the aerial part, Hg_s_ 7 mg kg^–1^ was the highest, with absorption of 49.46 mg kg^–1^, followed by Hg_s_ 5 (24.32), which was higher than the Hg_s_ of 0, 1, and 3 mg kg^–1^ (0.42, 4.04, and 3.39, respectively), which did not differ significantly from each other. In the roots, for the Hg_s_ of 7 mg kg^–1^ the concentration of Hg absorbed was 117.63 mg kg^–1^, statistically higher than the concentration of Hg absorbed at Hg_s_ 0, 1, 3 and 5 (0.28, 2.58, 16.21, and 34.59, respectively), which did not differ significantly from each other.

### 3.3 Phytoremediation Indices

The BCF values varied between the Hg_s_, (ANOVA, F = 43.36, *p* < 0.05), suggesting that the Hg_s_ applied had a direct influence on bioaccumulation. The Scott-Knott test indicated that the BCF averages for the different Hg_s_ concentrations differed statistically. There was variation in the BAF between the Hg_s_ studied (ANOVA, F = 32.62, *p* < 0.05), suggesting that the Hg_s_ applied directly influenced bioaccumulation. The results of the Scott-Knott test indicated the formation of three distinct groups between the different Hg concentrations, with average values of 12.75 for 7 mg kg^–1^, 1.76 for 3 mg kg^–1^, and an average of 9.41 for 1 and 5 mg kg^–1^. The TF values differed between the different Hg_s_ (ANOVA, F = 14.08, *p* < 0.05), forming four distinct groups. The TF, BAF, and BCF data can be seen in Table 4.

**Table 4:** Translocation, Bioconcentration, and Bioaccumulation Factors (Dry Weight Basis), and Mercury Concentration (mg kg^–1^) of *T. procumbens* as a Function of Hg Concentration in the Substrate^a^

	phytoremediation indices		
Hg_s_ (mg kg^–1^)	TF	BCF	BAF
0			
1	1.56 ± 0.04 ^d^	6.07 ± 0.02 ^a^	9.49 ± 0.01 ^b^
3	0.21 ± 0.01 ^a^	8.40 ± 0.05 ^b^	1.76 ± 0.02 ^a^
5	0.70 ± 0.02 ^c^	13.26 ± 0.04 ^c^	9.32 ± 0.02 ^b^
7	0.42 ± 0.02 ^b^	30.31 ± 0.04 ^d^	12.75 ± 0.02 ^c^

a Translocation factors (TF), bioconcentration factors (BCF), and bioaccumulation factors (BAF). Different lowercase letters in the columns indicate statistical differences by Scott-Knott (*p* < 0.05); equal letters do not differ significantly.

The mass of Hg removed by *T. procumbens* was assessed by the absorption index (AI) (Table 5). It can be seen that the mass of accumulated Hg varied significantly with increasing concentrations of Hg in the substrate (ANOVA, F = 48.96, *p* < 0.05).

**Table 5:** Absorption Index (AI) of *T. procumbens*, in μg of Hg, for the Different Concentrations of Hg in the Substrate^a^

absorption index (μg)			
Hg_s_ (mg kg^–1^)	whole plant	aerial	root
1	8.12 ± 0.79 ^a^	6.31 ± 0.53 ^b^	1.81 ± 0.08 ^a^
3	7.48 ± 0.67 ^a^	2.48 ± 0.23 ^a^	4.99 ± 0.48 ^b^
5	17.81 ± 1.37 ^c^	6.35 ± 0.61 ^b^	11.46 ± 1.18 ^c^
7	10.52 ± 1.03 ^b^	4.66 ± 0.36 ^c^	5.85 ± 0.56 ^b^

a Different lowercase letters in the columns indicate statistical differences using the Scott-Knott test (*p* < 0.05); equal letters do not differ significantly from one another.

The Scott-Knott test applied to the AI of T. procumbens indicated that for the whole plant, the averages of the absorption index formed three distinct groups, with the Hg_s_ of 5 mg kg^–1^ showing the highest accumulation index (17.81 μg) and distinguished from the Hg_s_ of 7 mg kg^–1^ (10.52 μg) and the Hg_s_ of 1 mg kg^–1^ (8.12 μg) and 3 mg kg^–1^ (7.48 μg), which are equal to each other and different from the others.

For the root, the test showed that for the Hg_s_ of 1 mg kg^–1^, the AI was 1.81 μg and differed from the Hg_s_ of 5 mg kg^–1^ (11.46 μg) and the Hg_s_ of 3 mg kg^–1^ (4.99 μg) and 7 mg kg^–1^ (5.58 μg), which were also similar to each other but formed a group distinct from the others. In the aerial part, the absorption indices for Hg_s_ of 1 and 5 mg kg^–1^ were 6.31 and 6.35 μg, which did not differ from each other and formed a group with higher values than those obtained with Hg_s_ of 7 mg kg^–1^ (4.66 μg), which, in turn, was higher than the concentration of 3 mg kg^–1^ (2.48 μg).

## 4 Discussion

The various parameters discussed throughout this study were important for better understanding the characteristics, behavior, and potential of *T. procumbens* or phytoremediation of Hg-contaminated soils. It was observed that the presence of Hg inhibited the plant’s growth, and it can be said that *T. procumbens* had a harder time growing in substrates contaminated with higher concentrations of the metal. The relative elongation ratios corroborate the evidence that the plant had difficulty establishing itself under higher concentrations of Hg_s_, with a decrease in RER as a result of the increase in the contaminant in the substrate, showing a significant difference between the sampling units. The aerial RER showed a percentage reduction of 17% in the Hg_s_ of 3 mg kg^–1^ and 67% in the Hg_s_ of 5 and 7 mg kg^–1^, while the root RER showed a percentage reduction of 54% in the Hg_s_ of 3, 5, and 7 mg kg^–1^.

### 4.1 Elongation and Biomass Production

The specimens cultivated in Hg_s_ of 7 mg kg^–1^ suffered a reduction in biomass of approximately 93.40%. This reduction was also significant for Hg_s_ of 3 and 5 mg kg^–1^, with biomass production on average being 61.79% lower. The decrease in biomass production was significant from a Hg_s_ of 3 mg kg^–1^, reaching its maximum point at an Hg_s_ of 7 mg kg^–1^. In studies with the species *Mentha arvensis* L. (Lamiaceae)^42^ and *Medicago sativa L.* (Fabaceae),^43^ it was observed that these plants were unable to demonstrate tolerance to Hg at levels similar to those used in this study. However, *T. procumbens* was able to develop in a way similar to that of the control when Hg_s_ was a maximum of 1 mg kg^–1^, as well as maintaining a TI value of 47.29% at 3 mg kg^–1^.

The data presented in this study indicate that *T. procumbens* can be classified as a phytoextractor plant, even under conditions of growth inhibition, such as those observed at Hg_s_ of 5 and 7 mg kg^–1^, since the low biomass production in these treatments was totally or partially compensated by the high capacity for accumulating Hg throughout the plant, as recorded by the increases in BAF, BCF and AI. Thus, it can be suggested that the plant is an alternative for phytoremediation of Hg-contaminated soils. Further studies are needed on the applicability of *T. procumbens* at soil contamination levels starting at Hg_s_ of 7 mg kg^–1^, with the need for differentiated management at levels similar to Hg_s_ of 5 and 7 mg kg^–1^, due to growth inhibition.

### 4.2 Tolerance Index

*Tridax procumbens* showed sensitivity to Hg, germinating and showing different developmental variations depending on the concentration of Hg_s_. *Tridax procumbens* proved to be partially tolerant to the Hg-contaminated substrate, with tolerance to Hg_s_ of 1 mg kg^–1^, being sensitive at Hg_s_ values of 3 and 5 mg kg^–1^ and highly sensitive at Hg_s_ and 7 mg kg^–1^. The TI is established by different levels of sensitivity according to the values obtained by the ratio between the dry biomass of the control plant and the other treatments, so a TI ≤ 25% is classified as highly sensitive, TI between 25 and 50% as sensitive, TI between 50 and 75% as moderate tolerance, TI between 75 and 100% considered tolerant, and TI > 100% classified as highly tolerant.^37^

These indices allow us to infer that despite germinating and producing biomass even in the substrate with the highest concentration of Hg, the plant showed sensitivity to the contaminant, suggesting that its presence in the habitat indicates that the area is not contaminated with more than 7 mg kg^–1^ of Hg in the soil. Despite the lower development according to the treatment adopted, *T. procumbens* did not show chlorosis or necrosis, as did the phytoremediator *Jatropha curcas L. (Euphorbiaceae)* ^44^ and unlike *Cyrtomium macrophyllum* (D. Don) Ching (Dryopteridaceae)^45^ and *Arabidopsis thaliana* (L.) Heynh. (Brassicaceae),^44^ which suffered these toxic effects in their plant tissues.

Although this study did not investigate physiological or molecular mechanisms, it is possible that the observed tolerance is associated with antioxidant enzyme systems or metal-binding compounds, as described for other species under Hg stress. This includes the action of superoxide dismutase (SOD), catalase (CAT), and glutathione-related compounds, which contribute to oxidative stress mitigation and metal detoxification^46,47^

### 4.3 Accumulation of Hg in Plant Tissues

The concentration of Hg in *T. procumbens* was higher in the root part compared to the aerial part at Hg_s_ of 3, 5, and 7 mg kg^–1^ and higher in the aerial tissue at Hg_s_ of 1 mg kg^–1^. This trend of greater accumulation of Hg in the root zone was also observed for *Jatropha curcas* ^44^ and *Erato polymnioides* (L.) Sw. (Asteraceae).^48^ These results are consistent with studies showing that toxic metal concentrations are higher in the root part of the plant.^49,50^ This is related to the fact that the absorption of metals by the roots is faster, as it does not depend on the translocation process to other parts of the plant.^51^ This may be a preventative strategy for the plant, since the root system acts as the first line of defense, restricting the movement of contaminants to its other tissues.^51^

In this study, the concentration of Hg in the aerial parts and roots of *T. procumbens* increased exponentially with the concentration of Hg used in the treatment, which in turn was inversely proportional to biomass production, indicating that the growth and development of the plant were negatively sensitized by the concentration of Hg in the substrate. These results indicate that both the whole plant and its specific parts respond differently to Hg concentrations, with greater sensitivity at higher concentrations.

As found in this experiment with *T. procumbens*, studies have shown a significant positive correlation between the Hg accumulated in plant parts in relation to that present in the growing substrate.^19,44^ Only at Hg_s_ of 1 mg kg^–1^, was there a greater accumulation in the aerial part of the plant to the detriment of the root system. Although no signs of Hg toxicity were observed, such as necrosis, chlorosis, wilting, or death of the plants, there was an inhibition in total biomass production, showing that there is a tolerated limiting level of Hg for the development of this species at levels above the Hg_s_ of 3 mg kg^–1^.

Plants adapted to and capable of growing in soils rich in heavy metals are called metallophytes, and among metallophytes, those that absorb and accumulate high levels of heavy metals in their aerial parts are called metal indicators.^52^ The concentration of heavy metals in shoots and leaves serves as a reflection of the heavy metal content of the soil and due to their ability to absorb them. These indicator plants play a key role in signaling the possible presence of heavy metals in the environment.^53^ *Pteris vittata* L. (Pteridaceae)^54^ and *Artemisia lavandulaefolia* DC. (Asteraceae)^55^ are some of the metallophytic plants used in the bioindication of soil contamination by heavy metals. *T. procumbens*, due to its characteristics of high accumulation and tolerance to Hg, can be considered a metallophyte, contributing to the bioindication and phytoremediation of contaminated soils.

### 4.4 Phytoremediation Factors

*Tridax procumbens* has potential for phytoremediation of Hg-contaminated soils, with good biomass production up to the levels adopted in the Hg_s_ of 3 mg kg^–1^ and with great accumulation and reduction of biomass at levels similar to the Hg_s_ of 5 and 7 mg kg^–1^, a fact corroborated by the concentrations of Hg in the different plant tissues and by the TF, BCF, and BAF factors.^38^ BAF values <0.01 indicate that the plant is a nonaccumulator, values between 0.01 and 0.1 show that the plant is a low accumulator, values between 0.1 and 1 characterize it as a moderate accumulator and being within the range of values between 1 and 10, plants with TF and BCF indices greater than 1 (TF, BCF, and BAF > 1) are potential for the phytoextraction process, however, if the TF and BCF are less than 1 (FT and BCF < 1), the plant is classified as an excluder.^19,51,56^

The translocation factors did not differ statistically, but they did reach an average value (TF = 0.72). This TF value did not reach the threshold of TF > 1, which in principle would be essential for a phytoremediation plant, unlike the other factors measured, which exceeded this threshold. This difference is due to the different behavior of Hg accumulation in these tissues.^57^ Although it is a relevant coefficient from an experimental point of view in terms of plant behavior under Hg contamination, TF alone is not considered a preponderant factor in determining the phytoremediation capacity of a species, and should be evaluated according to the specifics of each scenario and species, as well as considering other indices relating to the absorption of the contaminant by the plant, such as BCF and BAF.^50^

The BAF and BCF indices obtained for *T. procumbens* in all treatments were greater than 1 (BAF and BCF > 1), with an average BAF of 11.04 for Hg_s_ of 5 and 7 mg kg^–1^ and 1.46 for Hg_s_ of 1 and 3 mg kg^–1^, a maximum value close to that found for *Petroselinum crispum* (Mill.) Nyman (Apiaceae).^58^ At Hg_s_ of 7 mg kg^–1^ a BCF of 30.31 was obtained, which was higher than the BCF value found for *Cytromium macrohyllum* (D. Don) Ching (Dryopteridaceae).^1,45^ *Pleurozium schreberi* (Brid.) Mitt. (Pottiaceae), a plant considered to be a bioindicator of Hg pollution in the soil, showed a BAF ranging from 1.5 to 6.7,^59^ with minimum values similar to the Hg_s_ of 1 and 3 mg kg^–1^, and maximum values 39.31% lower than those shown by *T. procumbens* at Hg_s_ of 7 mg kg^–1^.

The increase observed in BCF and BAF values was inversely proportional to the growth and development of the species, indicating that the greater the soil contamination and the proliferation of this contaminant in the plant’s tissues, the lower the biomass production in the degraded environment. Hg inhibits plant growth by compromising various physiological processes. It causes degradation of the photosynthetic system, alters the permeability of cell membranes, and induces oxidative stress through lipid peroxidation and the accumulation of reactive oxygen species. This results in damage to cell structures and a reduction in biomass production.^60^

### 4.5 Absorption Index

Calculating the absorption index (AI) measured the mass of Hg removed by the plant, demonstrating the ability of *T. procumbens* to accumulate metal in its tissues. It was observed that, even with lower biomass production for the 5 and 7 mg kg^–1^ Hg_s_, Hg absorption was significantly higher in these treatments. Absorption for the Hg_s_ of 5 mg kg^–1^ was 128.33% higher than the average for the 1 and 3 mg kg^–1^ Hg_s_, q, which was 7.80 μg. Absorption in the 5 mg kg^–1^ Hg_s_ was 90% higher than that in the 7 mg kg^–1^ Hg_s_. In turn, Hg absorption in the 7 mg kg^–1^ Hg_s_ was 34.87% higher than the average of the 1 and 3 mg kg^–1^ Hg_s_. These results indicate that, despite the lower biomass production, the treatments with 5 and 7 mg kg^–1^ Hg resulted in a greater efficiency of metal absorption by the plant. It is also important to note that the smaller plants grown under higher Hg concentrations absorbed a similar or even greater total mass of Hg compared to larger plants under lower contamination, reinforcing the species’ efficiency despite reduced biomass.

Although the 1 mg kg^–1^ Hg_s_ showed the highest tolerance to Hg according to the tolerance index (TI), its mass of Hg removed was the second lowest (IA = 8.12 μg), with a small difference compared to the 3 mg kg^–1^ Hg_s_ (AI = 7.48 μg), which showed a TI value of 47.49%, indicating sensitivity to the contaminant, with the AI values being statistically similar. The concentrations of 5 and 7 mg kg^–1^, although they showed TI of 28.08% (sensitive) and 6.72% (highly sensitive), achieved the highest values of Hg mass removed, with AI of 17.81 and 10.52 μg, respectively. It can therefore be seen that TI and AI were inversely proportional, highlighting the ability of *T. procumbens* to absorb the contaminant, even with its reduced biomass and high sensitivity to Hg.

The increase in the concentration of Hg in the biomass, observed for the highest concentrations of Hg_s_, suggests that TI should be used with caution when predicting the applicability of a plant species in the remediation of contaminated soils. In the contaminations where the Tl was lower, such as in the 5 and 7 mg kg^–1^ Hg_s_, the mass of Hg removed from the substrate by the biomass was substantially higher than that in the 1 mg kg^–1^ Hg_s_, which had a Tl classified as tolerant and biomass production similar to that of the control (0 mg kg^–1^).

This plant behavior suggests that changes in field management, such as successive plantings and harvests or increased growing time, can be effective strategies for phytoremediation of soils contaminated with Hg concentrations above 3 mg kg^–1^. This phytomanagement can include some guidelines, such as the selection of species that adapt to the site, evaluation of genetic material, field trials, valorization of biomass, use of amendments, and consideration of climatic factors for the stabilization of toxic metals, aiming for a self-sustaining and resilient ecosystem.^61^ However, even though the plant grew more stunted and with lower biomass production in the treatments with higher Hg concentrations, in general terms, *T. procumbens* behaved as a phytoremediator, maintaining the mass of Hg removed and extracting the contaminant from the soil, with excellent BAF and BCF values.

### 4.6 Potential for Phytoremediation and Bioindication

In view of the results obtained, it was found that *T. procumbens* showed a good ability to accumulate Hg, promoting its removal from the soil in a satisfactory manner, indicating phytostabilization and phytoextraction of the contaminant from the soil.^19,62,63^ The fact that it is characterized as a species with rapid proliferation and low nutrient and soil requirements, spontaneous and thrives in adverse conditions, even in the presence of mercury, indicates that it can be considered for soils with levels of contamination similar to those used in this study.

Furthermore, the present findings are consistent with those of dos Santos Soares et al.,^26^ who identified *T. procumbens* in urban environments with detectable levels of mercury in the soil, where it demonstrated the ability to absorb the metal even under noncontrolled conditions, reinforcing its potential as a bioindicator and supporting its selection for controlled experimental evaluation. However, *T. procumbens* may not show tolerance to higher concentrations of the contaminant in the soil beyond 7 mg kg^–1^.

*Tridax procumbens* can be used as a bioindicator of soil contamination by Hg, signaling the presence of this metal, both by the accumulation of Hg in the biomass and by the difficulty of growing in soils with Hg concentrations higher than 3 mg kg^–1^. Bioindicator species are used to check the quality of a specific environment, indicating the existence of contaminants or their quantity.^64^ Basic characteristics such as accessibility, bioecological suitability, reliability and representativeness are essential for a species to be recognized as a bioindicator.^18^ In terms of these prerequisites, *T. procumbens* can be considered a species suitable for bioindication of Hg-contaminated soils.

With regard to applications, the results obtained show that this species has potential for phytoremediation. *Tridax procumbens* is easy to grow and propagate and can be found in regions with different climates and soil conditions, which means that it can be applied as a phytoremediator of soil contamination by Hg. Its ability to germinate and spread also allows it to be applied to large areas. Although these findings are based on controlled greenhouse conditions, future studies under field scenarios are needed to confirm the phytoremediation efficiency of *T. procumbens* in real contaminated environments. Factors such as soil heterogeneity, microbiota interactions, and long-term contaminant dynamics should be evaluated to ensure ecological viability and practical applicability.

## 5 Conclusions

In this study, the phytoremediation potential of soil Hg was evaluated using *T. procumbens*, a spontaneous plant that is easy to reproduce and widespread, which showed tolerance to the contaminant. The plant’s development was inhibited as the Hg_s_, increased, while the AI increased as the Hg_s_ increased, indicating that even with the lower biomass production, the ability to absorb Hg from the substrate increased, making it useful for phytoremediation of soils contaminated with up to 7 mg kg^–1^ of Hg. The high rates of bioaccumulation and bioconcentration shown by the plant are credentials that confirm that *T. procumbens* is a promising phytoremediation species for Hg contamination in the soil. Under these conditions, the plants showed no signs of oxidative stress or toxicity in their plant tissues in any of the Hgs studied.

*Tridax procumbens* proved to be a bioindicator of Hg pollution, accumulating concentrations of the contaminant above the levels found in the soil in which it is grown, which makes it possible to identify sites degraded by Hg. The large accumulation of Hg by this species allows the contaminant to be detected in the soil, a fact that would be unlikely in the absence of the accumulating organism. Furthermore, its bioindicator profile is corroborated by the fact that accumulated Hg is easily detected in its plant tissues.

The levels of translocation, bioconcentration, and bioaccumulation of Hg were different between the treatments adopted in the experiment according to the contamination applied, in such a way that the plant demonstrated that it can be cultivated and used in the phytoremediation of soils with Hg concentrations similar to those in this study, showing characteristics such as tolerance to the contaminant and its accumulation in the plant’s tissues, ease of cultivation and management, good reproduction, and adaptation to different climate and soil conditions, which make it suitable for use as a phytoremediator and bioindicator of soils contaminated by this metal, with the ability to phytostabilize Hg in its roots and to perform phytoextraction of soil Hg, as it has a high capacity for accumulation in the tissues of roots and aerial parts.

## Data Availability

The data that support the findings of this study are available from the corresponding author upon reasonable request. Due to the double-blind review process, specific data access details cannot be provided at this stage.
